# [Corrigendum] ND‑09 inhibits chronic myeloid leukemia K562 cell growth by regulating BCR‑ABL signaling

**DOI:** 10.3892/or.2025.8870

**Published:** 2025-01-27

**Authors:** Yan-Hong Liu, Man Zhu, Pan-Pan Lei, Xiao-Yan Pan, Wei-Na Ma

Oncol Rep 46: 136, 2021; DOI: 10.3892/or.2021.8087

Subsequently to the publication of the above article, and a Corrigendum that has already been published with the intention of showing a corrected version of Fig. 2A (DOI: 10.3892/or.2023.8518; published online on March 3, 2023), the authors have subsequently realized that other errors were featured in certain of the published figures. First, the authors realized that Fig. 3 on p. 5 was incorrectly assembled: specifically, the flow cytometric data included in Figs. 3A and 3B were inadvertently assembled incorrectly. Additionally, the authors note that the control GAPDH data were duplicated in Figs. 5A and 5B, 6A and 6B, and 7A and 7B on p. 7 and 8. These errors occurred on account of the fact that the authors chose to use the same GAPDH bands for quantitative analysis, which they subsequently realize was not an appropriate course of action; the original GAPDH bands pertaining to the correct experiments are now included in each of these figures.

The revised versions of Figs. 3, 5, 6 and 7 are shown on the next two pages. Note that the revisions made to Figs. 3, 5, 6 and 7 in this paper do not have a major impact on the reported results, and neither do they affect the overall conclusions reported in the study. All the authors agree to the publication of this corrigendum. The authors are grateful to the Editor of *Oncology Reports* for allowing them the opportunity to publish this additional Corrigendum; furthermore, they apologize for any inconvenience caused to the readership of the Journal.

## Figures and Tables

**Figure 3.**
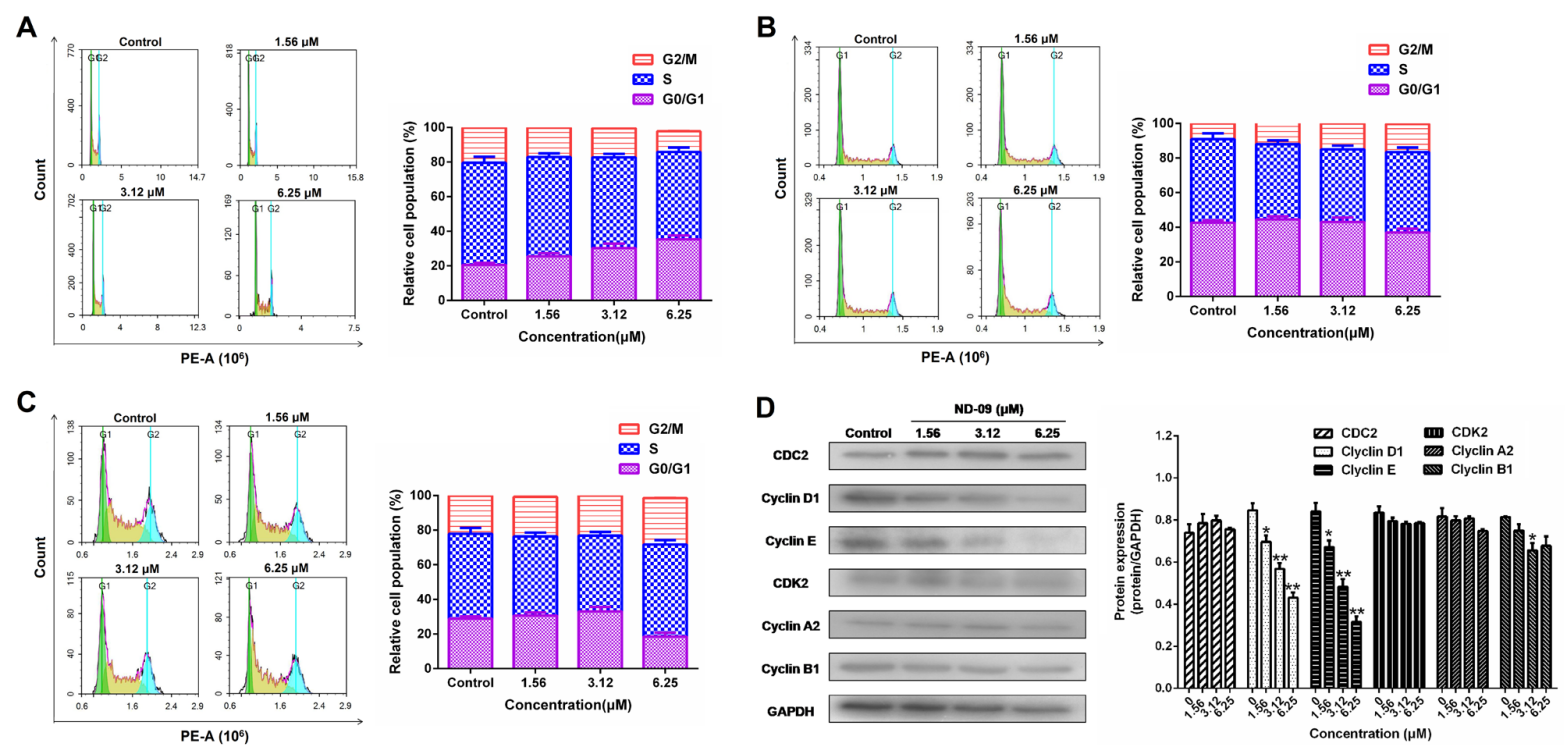
Effect of ND-09 treatment on the cell cycle. Representative flow cytometry DNA content histogram of (A) K562, (B) JURKAT, and (C) HUT78 cells after treatment with ND-09 (0, 1.56, 3.12 and 6.25 µM). (D) Effects of ND-09 on cell cycle-related protein expression in K562 cells. All results were quantified by densitometric analysis of the bands and were normalized to GAPDH (internal control). Samples were derived from the same experiment, and blots were processed in parallel. Values represent the average of three independent experiments. Data are presented as mean ± SEM (n=3). *P<0.05, **P<0.01 compared to the untreated control group.

**Figure 5.**
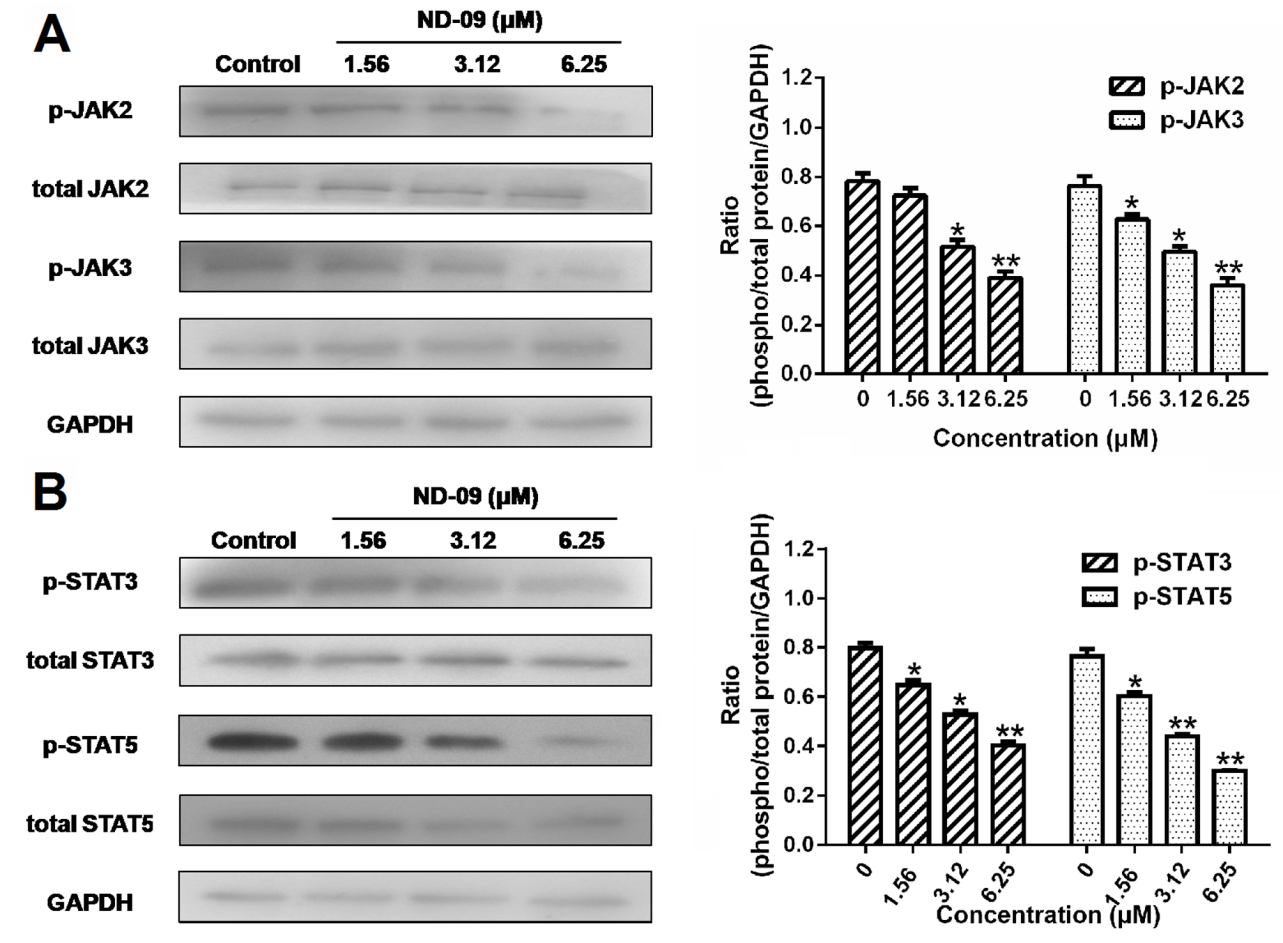
Effects of ND-09 on JAK/STAT signaling protein levels. (A) Protein levels of JAK2, p-JAK2, JAK3, and p-JAK3 in K562 cells treated with ND-09 were evaluated by western blot analysis. (B) Protein levels of STAT3, p-STAT3, STAT5, and p-STAT5 in K562 cells treated with ND-09 were evaluated by western blot analysis. Results were quantified by densitometry analysis of the bands and were normalized to GAPDH (internal control). Samples were derived from the same experiment, and blots were processed in parallel. Values represent the average of three independent experiments. Data are presented as mean ± SEM (n=3). *P<0.05, **P<0.01 compared to the untreated control group.

**Figure 6.**
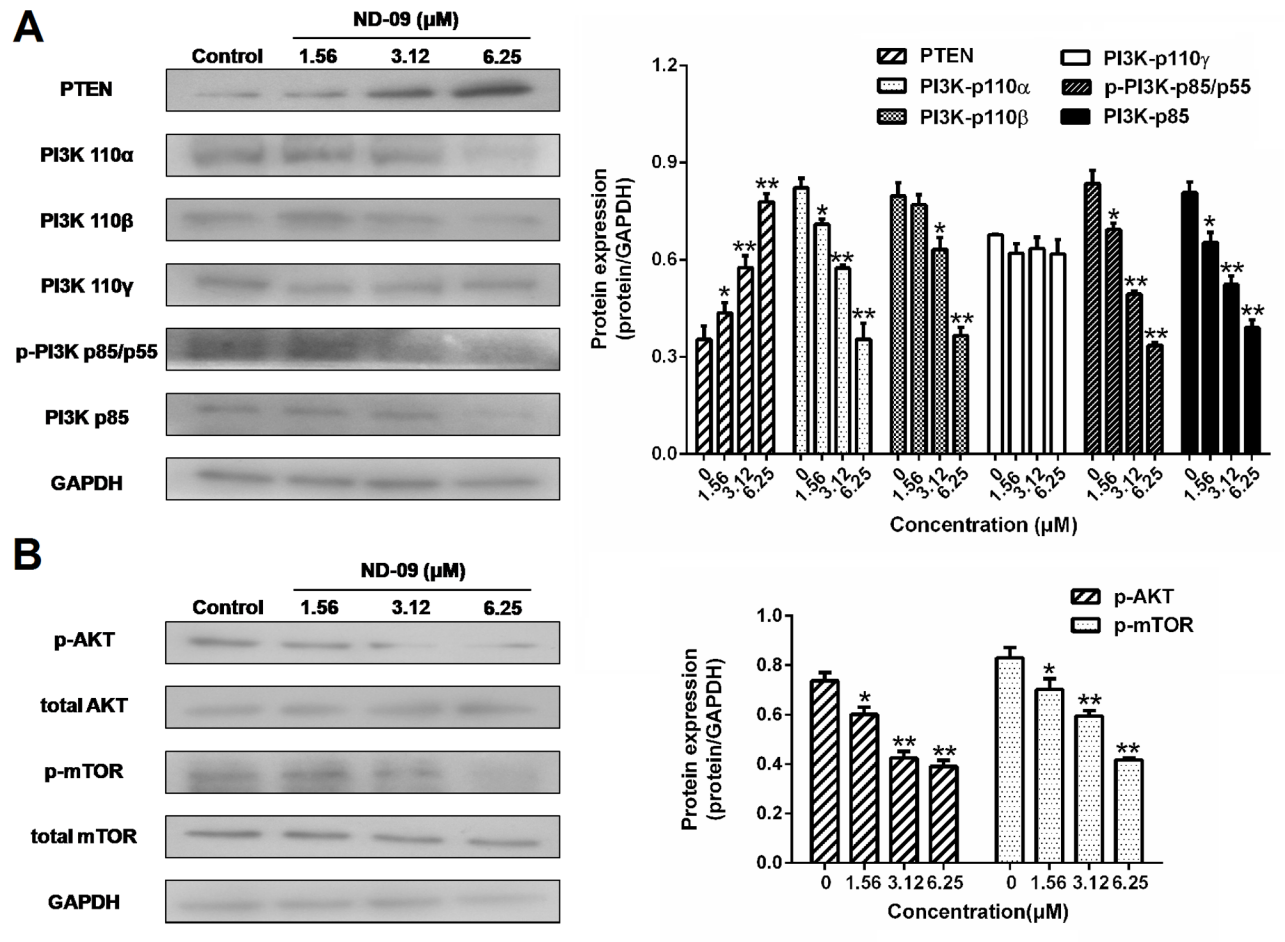
Effects of ND-09 on PI3K/AKT signaling protein levels. (A) Protein levels of PTEN, PI3K-p110α, PI3K-p110β, PI3K-p110γ, p-PI3K p85/p55, and PI3K-p85 in K562 cells treated with ND-09 were evaluated by western blot analysis. (B) Protein levels of AKT, p-AKT, mTOR, and p-mTOR in K562 cells treated with ND-09 were evaluated by western blot analysis. Results were quantified by densitometric analysis of the bands and were normalized to GAPDH (internal control). Samples were derived from the same experiment, and blots were processed in parallel. Values represent the average of three independent experiments. Data are presented as mean ± SEM (n=3). *P<0.05, **P<0.01 compared to the untreated control group.

**Figure 7.**
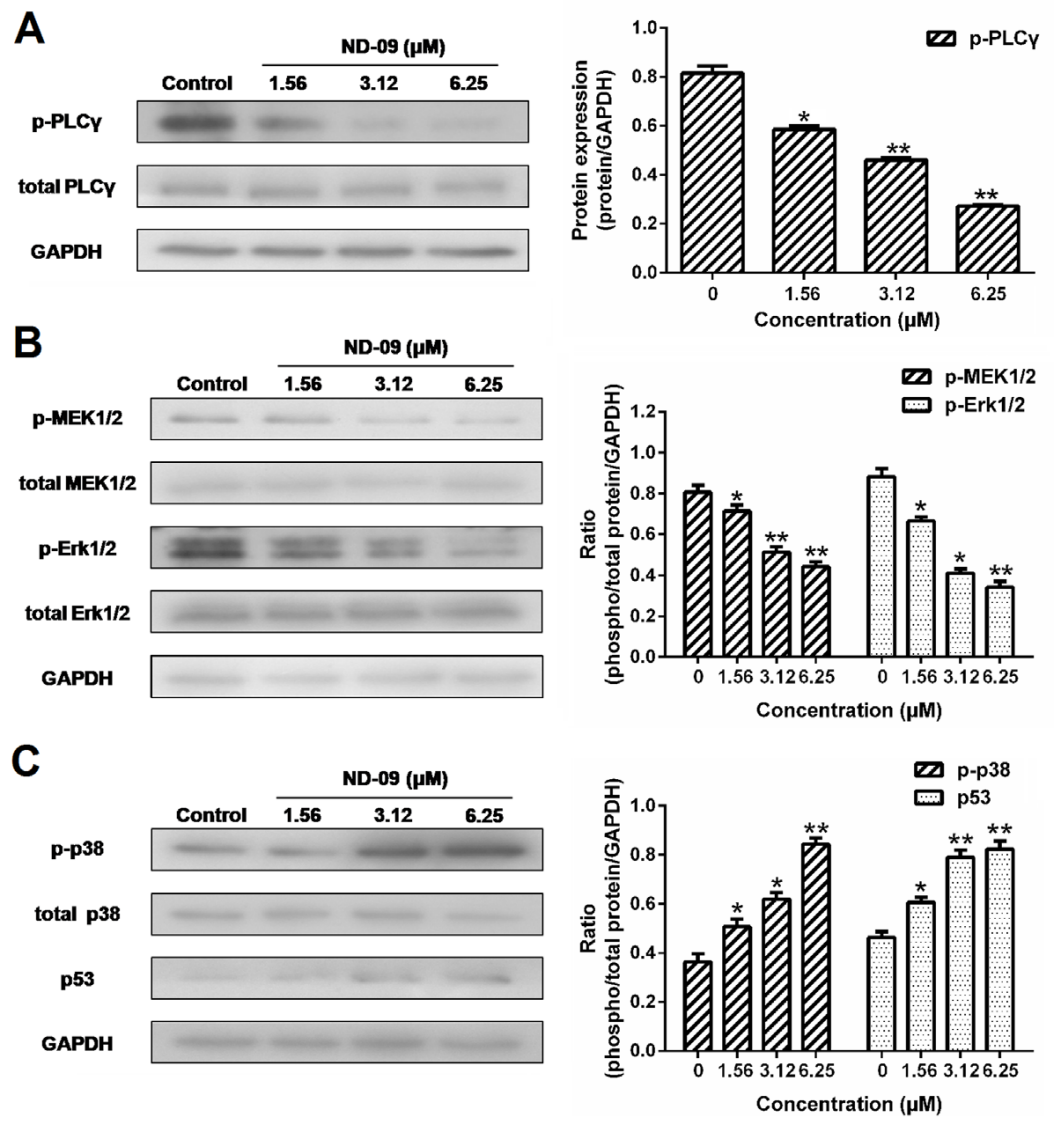
Effects of ND-09 on MAPK signaling protein levels. (A) Protein levels of PLCγ and p-PLCγ in K562 cells treated with ND-09 were examined by western blot analysis. (B) Protein levels of MEK1/2, p-MEK1/2, Erk1/2, and p-Erk1/2 in K562 cells treated with ND-09 were examined by western blot analysis. (C) Protein levels of p38, p-p38, and p53 in K562 cells treated with ND-09 were examined by western blot analysis. Results were quantified by densitometric analysis of the bands and were normalized to GAPDH (internal control). Samples were derived from the same experiment, and blots were processed in parallel. The values represent the average of three independent experiments. Data are presented as mean ± SEM (n=3). *P<0.05, **P<0.01 compared to the untreated control group.

